# Advances and challenges in poliomyelitis vaccines: a comprehensive review of development, production, and global deployment

**DOI:** 10.3389/fpubh.2025.1611028

**Published:** 2025-07-16

**Authors:** Jia Liang, Qi Zhang, Yanan Li, Lili Wang

**Affiliations:** ^1^Beijing Institute of Biological Products, Co., Ltd., Beijing, China; ^2^Hebei Provincial Children's Hospital, Shijiazhuang, China; ^3^Department of Anesthesiology, The Third Hospital of Hebei Medical University, Shijiazhuang, China

**Keywords:** poliomyelitis vaccines, inactivated poliovirus vaccine, oral poliovirus vaccine, vaccine-derived polioviruses, global polio eradication initiative, vaccine production

## Abstract

Poliomyelitis has been a significant global health challenge for centuries. Since the launch of the Global Polio Eradication Initiative (GPEI) in 1988, remarkable progress has been achieved, with wild poliovirus (WPV) cases reduced by over 99%. However, challenges persist, including endemic transmission in conflict zones, the emergence of vaccine-derived polioviruses (VDPVs), and the complex logistics of vaccine production and distribution. This review synthesized the latest advancements in poliovirus vaccine development, production, and global deployment. Specific topics include the historical milestones of inactivated poliovirus vaccine (IPV) and oral poliovirus vaccine (OPV), innovations in next-generation vaccines such as novel OPV (nOPV2), intradermal IPV (IIPV), virus-like particle (VLP) vaccines, and mRNA vaccines, as well as critical considerations in manufacturing, quality control, and regulatory compliance. We also examined global strategies for vaccination campaigns, cold chain management, and eradication-endgame planning, alongside emerging challenges like VDPVs outbreaks, funding constraints, and geopolitical barriers. The significance of sustained global cooperation, equitable resource allocation, and technological advancement are essential to achieving a polio-free world, with the integration of scientific innovation with public health strategies. The lessons and insights presented herein inform polio eradication efforts, providing a blueprint for future disease eradication initiatives. The importance of resilience, adaptability, and community engagement was also emphasized for global health governance.

## Introduction

Poliomyelitis, a highly infectious disease caused by a virus belonging to the *Picornaviridae* family, has been a long-standing global public health menace (1). Historically, it was associated with crippling deformities, affecting thousands of lives worldwide. There are three distinct serotypes of the poliovirus: serotype 1 (*PV-1*), serotype 2 (*PV-2*), and serotype 3 (*PV-3*). *PV-1* is the predominant type associated with paralysis and is the primary cause of epidemics. It remains the only wild poliovirus type currently circulating, though its prevalence has been significantly reduced through global vaccination efforts. The virus primarily spreads through the fecal-oral route, though respiratory transmission can also occur. The clinical features range from mild respiratory illness, gastroenteritis, and malaise to severe paralytic forms. These are categorized into inapparent infection, mild illness (abortive poliomyelitis), aseptic meningitis (non-paralytic poliomyelitis), and paralytic poliomyelitis.

The development of the Salk inactivated poliovirus vaccine (IPV) and Sabin oral poliovirus vaccine (OPV) were significant milestones in the fight against poliomyelitis. The IPV, developed by Jonas Salk in the 1950s, was the first polio vaccine to be widely used. IPV stimulates a systemic immune response, providing protection against paralytic poliomyelitis. The OPV, developed by Albert Sabin in the 1960s, contains live attenuated poliovirus strains. These vaccines have the advantage of inducing humoral and mucosal immunity, which is crucial for preventing the spread of poliovirus through the fecal-oral route. OPV is also more cost-effective, making it suitable for mass vaccination campaigns in resource-limited settings.

Since the launch of the Global Polio Eradication Initiative (GPEI) in 1988 by the World Health Organization (WHO), vaccination campaigns have had a profound impact on global polio incidence. The use of OPV has played a central role in this process, while the introduction and application of IPV have provided supplementary support. By 2019, the use of OPV and the introduction of IPV in many countries have led to more than 99% reduction (only 125 cases) from 1988 in the number of polio cases globally (2). For example, in India, the intensive use of vaccination campaigns led to the elimination of wild poliovirus, and the country was certified polio-free in 2011, followed by the entire South-East Asia Region in 2014. However, wild poliovirus (WPV) remains in some regions with low vaccination coverage, such as parts of Afghanistan and Pakistan. The two countries share a long border with extensive population movement, which complicates the eradication efforts as the virus can easily spread across the border (3).

Notably, a major concern with OPV is the rare occurrence of vaccine-associated paralytic poliomyelitis (VAPP). Moreover, the vaccine-derived polioviruses (VDPVs) have emerged in African countries such as Malawi and Mozambique due to genetic reversion of the attenuated strains during replication in the human gut (4, 5). These are strains that have regained neurovirulence during replication in under-immunized individuals. As of July 2023, there were 12 cases of paralysis caused by WPV1 and 491 cases of paralysis caused by VDPVs reported globally (6), highlighting the ongoing challenges in achieving complete eradication. Accordingly, mass vaccination campaigns, including supplementary immunization activities (SIAs), are still essential in increasing population immunity. Concurrently, high-quality monitoring and the development of effective strategies to address the remaining low-immunity areas are critical to the success of GEPI (7).

## Scientific advances in poliovirus vaccines

### First-generation vaccines

#### Inactivated poliovirus vaccine (IPV)

IPV mainly consists of inactivated poliovirus and excipients (such as salts, buffers, adjuvants, trace amounts of antibiotic residues, etc.). For example, sodium dihydrogen phosphate and sodium hydrogen phosphate are used as buffers to maintain the pH stability of vaccines. Sodium chloride is used to regulate the osmotic pressure of vaccines. Aluminum hydroxide adjuvants adsorb antigens in vaccines, prolonging their retention time in the body and enhancing the uptake and processing of antigens by immune cells. Potassium chloride is typically used to maintain normal cellular physiological states and metabolic activities, thereby ensuring normal viral replication within cells. Gentamicin sulfate or kanamycin sulfate are used during vaccine production to inhibit the growth of contaminants, ensuring the sterility of the vaccine production environment, preventing contamination by other harmful microorganisms, and ensuring vaccine quality and safety. These antibiotic components are generally removed as much as possible during the vaccine purification process.

In the context of immunization against poliomyelitis, IPV plays a crucial role in generating a comprehensive immune response. Primarily, the immune system synthesizes IgM antibodies as an initial response to the vaccine (8). These antibodies are the first line of defense and are crucial in the early stages of immune response. Subsequently, the immune system generates IgG antibodies, which are instrumental in providing long-term immunity (9, 10). IPV also stimulates the production of neutralizing antibodies, which prevent the virus from entering host cells (11). Additionally, secretory IgA antibodies are produced at mucosal surfaces to block viral entry. Furthermore, memory B cells are generated, which can rapidly produce antibodies upon re-exposure to the virus (12, 13). These antibodies work together to enhance the body’s immune defense against poliovirus.

The production of IPV traditionally involves the cultivation of poliovirus in cell culture systems, with Vero cells being one of the most used substrates (14). The process begins with the cultivation of Vero cells on microcarriers in bioreactors, which allows for high-density cell growth and efficient virus production. Once the poliovirus has been propagated to the desired titer, it is subjected to inactivation, typically using formaldehyde, to ensure that the virus is no longer infectious while retaining its immunogenic properties (15, 16). But the approach has several drawbacks. Biologically, it involves using virulent strains, posing a risk of accidental viral release. This necessitates production in a biosafety level 3 laboratory with strict air flow control and emission disinfection to prevent contamination. There’s also a risk of viral mutation during culture, potentially affecting vaccine safety and efficacy. Economically, the process is costly due to the need for complex culture media and high-standard facilities. Additionally, the cell culture system’s low production efficiency limits virus yield, and the required purification and inactivation steps further increase production costs. Recent advancements in cell culture technology have introduced alternative platforms such as the PER. C6® cell line, which was used for high-yield production of poliovirus under serum-free conditions. The platform has shown promise in producing poliovirus with higher titers and D-antigen content compared to traditional Vero cell cultures. The PER. C6® cell line supports the replication of all three poliovirus serotypes (*PV-1*, *PV-2*, *PV-3*), offering a potential low-cost option for large-scale IPV production (17, 18).

In addition to traditional methods, there is ongoing research into optimizing IPV production with alternative virus strains and cell lines. For instance, the use of attenuated Sabin strains for IPV production is being explored as a safer alternative to wild-type strains. These strains, when grown on platforms like PER. C6®, have demonstrated high immunogenicity and safety in preclinical models, suggesting their potential for future IPV formulations (19, 20). Furthermore, innovations in cell culture techniques have addressed concerns related to the use of animal-derived components in vaccine production, such as the development of serum-free and chemically defined media. These advancements not only enhance the safety profile of the vaccine but also contribute to the consistency and scalability of the production process (21, 22).

Overall, the evolution of IPV production technologies reflects a concerted effort to improve the safety, efficacy, and affordability of polio vaccines, which are crucial for the global eradication of poliomyelitis. As research continues, these innovations hold the promise of more efficient and sustainable vaccine production methods.

#### Oral poliovirus vaccine (OPV)

OPV offers several advantages, including the induction of mucosal immunity, which is important for preventing poliovirus replication in the gut and subsequent transmission. Mucosal immunity is crucial because it provides the first line of defense at the entry points of pathogens, particularly in the gastrointestinal tract where poliovirus initially replicates. This type of immunity is characterized by the production of secretory IgA antibodies neutralizing pathogens at mucosal surfaces and preventing them from establishing infection and spreading further into the body. When a vaccinated person encounters WPV, the mucosal immune response could prevent the virus from replicating and being shed in feces, thereby reducing transmission to others. This characteristic of OPV has been instrumental in the global efforts to eradicate polio, with assisting to interrupt the chain of transmission in communities. Moreover, live attenuated vaccines could provide cross-protection against other pathogens by stimulating a broad immune response. The viral interference induced by live attenuated virus vaccines prevented infections such as otitis media caused by non-polio virus in clinical trial (23). This phenomenon underscores the potential of OPV to confer additional health benefits beyond its primary target.

The production of oral vaccines that induce mucosal immunity could be achieved through various cost-effective platforms beyond traditional methods. Plant-based systems offer a promising approach. Transgenic plants express vaccine antigens, such as certain rice and corn varieties. These plants provide a low-cost and scalable production system and protect antigens from gastric acid degradation due to their robust cell walls, enabling antigen delivery to the intestines and interaction with gut-associated lymphoid tissue to induce mucosal immunity. It was shown that the efficient expression of cholera toxin B subunit (CTB) in potato varieties using *Agrobacterium*-mediated transformation, establishing a scalable plant-based vaccine production platform (24). These transgenic potatoes serve as a safe and stable oral vaccine carrier, offering a cost-effective and cold-chain-free immunization solution for resource-limited settings. Microalgae present another innovative platform. With high photosynthetic efficiency and the ability to perform complex protein folding and modification, microalgae can produce active antigen proteins. They have short cultivation cycles, low production costs, and scalability advantages over bacterial and mammalian cell systems. Moreover, microalgae-derived proteins can be directly consumed without purification, further reducing costs. Research in mice models has indicated that algae-produced SARS-CoV-2 RBD induced systemic and mucosal humoral immune responses via oral administration, comparable to injected antigens with aluminum adjuvants, and the induced antibodies show similar reactivity against Delta and omicron variants (25). Future applications of these technologies could potentially be applied to the production of OPV.

#### Next,-generation vaccines

##### Novel OPV (nOPV2)

The emergence of VDPVs is a major concern, as they could cause paralytic poliomyelitis outbreaks in populations with low immunity (4). The novel OPV (nOPV2) has been engineered to improve the genetic stability of the Sabin oral poliovirus vaccine and reduce the emergence of VDPVs. The development of nOPV2 aims to maintain the immunogenic benefits of the original vaccine while significantly reducing the potential for these adverse events.

In a phase 3, double-blind, randomized controlled trial in The Gambia, nOPV2 was found to be immunogenic and safe in infants and young children. The seroconversion rates for the three lots of nOPV2 in infants ranged from 48.9 to 49.2%, and no safety concerns were identified (26). Furthermore, the study found that the post-two-dose seroprotecting rates were high, with no significant safety concerns identified. These results are pivotal for the licensure and WHO prequalification of nOPV2, as they provide robust evidence of its efficacy and safety profile. In Nigeria, the use of nOPV2 in outbreak response has been evaluated. A Bayesian time-series model showed that both nOPV2 and monovalent oral polio vaccine type 2 (mOPV2) campaigns were highly effective in reducing the transmission of VDPVs, on average reducing the susceptible population by 42% (95% CI 0.28–0.54) and 38% (95% CI 0.20–0.51) per campaign, respectively (27). These findings underscore the potential of nOPV2 in global polio eradication efforts by offering a more stable and reliable vaccine option.

#### Intradermal IPV

Intradermal IPV (IIPV) offers a dose-sparing strategy for resource-limited settings. A study in rats showed that intradermal delivery of fractional-dose Sabin-derived IPV combined with diphtheria-tetanus-acellular pertussis vaccine (DTaP-sIPV) using a hollow microneedle device (MicronJet600) had a significant dose-sparing effect. It induced more effective protection against *Bordetella pertussis* infection by causing Th1/Th17 responses compared to full-dose intramuscular immunization (28).

IIPV presents a promising dose-sparing strategy, particularly beneficial for resource-limited settings. By delivering a fractional dose of IPV intradermally, it is possible to stretch the limited global IPV supply while maintaining or even enhancing population immunity. This approach involves administering one-fifth of the full vaccine dose intradermally, which has been shown to elicit comparable immunogenic responses to the full intramuscular dose. The use of novel intradermal devices, such as intradermal adapters and disposable-syringe jet injectors, further facilitates this method, offering an efficient alternative to traditional needles and syringes.

The operational feasibility of implementing intradermal fractional dose IPV (fIPV) vaccination on a large scale has been demonstrated in countries like India, Pakistan, and Sri Lanka. These early-adopter countries have shown that it is possible to integrate fIPV into routine immunization and supplementary immunization activities effectively. The scientific data supporting this strategy, coupled with its operational success, encourages other countries to consider adopting fIPV as part of their immunization programs. This strategy not only addresses the issue of vaccine shortages but also contributes to the broader goal of polio eradication by ensuring that more individuals receive immunization coverage (29).

Another study in Bangladesh evaluated different vaccination schedules with intradermal fractional-dose IPV. It was found that although the immunogenicity of one-fifth fractional-dose IPV was inferior to that of full-dose IPV, the former still showed some priming effects, indicating its potential use in resource-limited settings where vaccine supply may be constrained (30).

#### Virus-like particles (VLPs)-based vaccines

The VLPs-based vaccines have emerged as a promising platform for the development of next-generation vaccines against poliovirus. These vaccines are designed to mimic the structure of the virus without containing its genetic material, thereby offering a safer alternative to traditional vaccines. The production of poliovirus VLPs involves the expression of viral capsid proteins in various expression systems, such as yeast and insect cells, which allows for the assembly of particles that are antigenically like the native virus. This approach has shown potential in preclinical studies, where VLPs have demonstrated the ability to induce strong immune responses comparable to those elicited by inactivated polio vaccines (31, 32). A study on chimeric VLPs targeting cutaneous human papillomaviruses demonstrated that immune sera cross-neutralized different virus types, indicating the potential of VLPs to induce broad-spectrum immunity (33). Although there are currently no VLPs-based polio vaccines in clinical use, the technology holds great potential for the development of next-generation polio vaccines, especially in terms of safety and the ability to induce strong immune responses.

One of the key advantages of VLPs-based poliovirus vaccines is safety profile. Unlike live-attenuated vaccines, VLPs cannot revert to a pathogenic form and eliminate the need for handling live virus during production, thus reducing biosafety concerns. Recent advancements in VLPs technology have focused on improving the stability and immunogenicity of these particles. For instance, stabilized VLPs produced in yeast have shown enhanced thermostability and the ability to elicit neutralizing antibodies in animal models, making them a viable candidate for future polio vaccination strategies (34, 35). The development of VLPs also benefits from the broader advancements in technology, which have been applied to various viral pathogens. The versatility of VLPs as a vaccine platform is highlighted by their successful use in vaccines against hepatitis B and human papillomavirus, as well as their ongoing development for other viral infections such as hepatitis C and respiratory viruses (36, 37). These successes underscore the potential of VLPs to provide broad protection against a range of diseases, including poliovirus. Furthermore, the use of VLPs in vaccine development is supported by their ability to induce both humoral and cellular immune responses. This dual activation of the immune system is crucial for effective protection against viral infections. The structural properties of VLPs, which include their repetitive surface geometry and size, contribute to their high immunogenicity and ability to mimic natural viral infections (38, 39).

In conclusion, the VLPs-based vaccines for poliovirus represent a promising avenue for achieving polio eradication in future. The ongoing research and development efforts are focused on optimizing the production processes, enhancing the immunogenicity, and ensuring the stability of these vaccines to meet the global demand for safe and effective polio immunization.

#### mRNA vaccines

The mRNA vaccines have exhibited great potential in the fight against viral diseases, and they also hold promise for poliovirus vaccination. The mRNA vaccines could be rapidly developed and produced in response to emerging viral threats. They work by encoding the viral antigen, which is then expressed by the host cells, triggering an immune response.

Compared to traditional vaccines, mRNA vaccines offer several advantages. They are designed to target specific epitopes, potentially leading to more targeted and effective immune responses. Additionally, the production process is relatively simple and can be scaled up quickly. However, challenges such as mRNA stability, delivery systems, and long-term safety need to be addressed before their widespread use for poliovirus vaccination (40).

The potential of mRNA vaccines in combating viral diseases and application in poliovirus vaccination highlight the transformative impact of this technology on public health. As research continues to advance, the mRNA vaccines are poised to play a crucial role in the prevention and control of infectious diseases worldwide, offering new way to address both existing and emerging health challenges.

## Production and quality control

### Manufacturing processes

#### Cell culture systems for poliovirus propagation

Vero cells are derived from the kidney cells of African green monkeys commonly used for poliovirus propagation. Vero cells support the replication of poliovirus to high titer. It has been reported that *PV3* was cultured using a suspension culture of Vero cell lines, resulting in a 30% increase in *PV3* viral-titer (41). This indicated that Vero cell lines cultured in suspension effectively support high-titer replication of poliovirus, providing an efficient cell substrate option for vaccine production.

The growth of Vero cells could be optimized using different cultivation methods. Semi-batch, perfusion, and recirculation strategies for media refreshment have been compared with batch cultivation. Increased cell densities achieved through these alternative methods allowed up to 3 times higher D-antigen levels when compared with batch-wise Vero cell culture, potentially reducing the vaccine cost per dose (16).

#### Challenges in IPV production

In IPV production, viral inactivation is a critical step. Complete inactivation of the poliovirus is essential to ensure the safety of the vaccine. However, over-inactivation leads to a loss of immunogenicity. Antigen standardization is also challenging, as it is necessary to ensure consistent antigen content and quality across different batches. Scaling up IPV production is difficult due to the complex manufacturing process and the need for high level containment facilities (14).

One promising method for viral inactivation is the use of electron beam (eBeam) irradiation. This technology has been successfully applied in other industries and attended in the pharmaceutical field for its ability to inactivate pathogens while preserving the antigenicity of the virus. This preservation is crucial to produce effective vaccines, as it ensures the immune system recognize and respond to the inactivated virus. A study demonstrated the effective use of eBeam irradiation for the inactivation of human rotavirus, which resulted in the production of neutralizing egg yolk antibodies. The antigenicity of the eBeam-inactivated virus was better preserved compared to thermally and chemically inactivated viruses, making it a suitable method for both passive and active immunization strategies (42).

Another approach to viral inactivation involves the use of photo-inactivation with non-nucleoside reverse transcriptase inhibitors (NNRTIs). This method has been shown to achieve complete inactivation of HIV-1 viral particles in suspension. By using a photoactive compound and exposing the viral particles to UV light, researchers were able to inactivate the virus while maintaining the conformational and antigenic integrity of viral surface proteins. This method allows for the large-scale production of inactivated viral particles, which is essential for vaccine development (43).

Additionally, continuous in-line viral inactivation processes are being developed to improve the efficiency and safety of monoclonal antibody therapeutics. Traditional viral inactivation methods involve maintaining the product at a low pH for a defined period, but continuous processing offers a more streamlined approach. A lab-scale prototype system has been designed to evaluate the kinetics of virus inactivation under various conditions, indicating comparability between continuous and traditional batch-mode viral inactivation (44).

These advancements in viral inactivation techniques are crucial for the development of safe and effective vaccines, including IPV, and highlight the importance of ongoing research in this field.

#### OPV production

In OPV production, the attenuation process is crucial. Attenuation refers to the process of weakening the virus so that it no longer causes disease in humans, yet it still elicits a strong immune response. The goal is to produce a vaccine strain that is immunogenic but not pathogenic, thereby providing immunity without causing the disease itself. The Sabin strain is produced by serial passages in different cell lines (such as monkey kidney cells), allowing the virus to gradually adapt to the cellular environment and reduce its tropism and pathogenicity for the human nervous system. OPV based on the Sabin strain has good immunogenicity and safety. However, ensuring batch-to-batch consistency in the attenuation process is challenging. Variations in the attenuation of the vaccine strains can lead to differences in immunogenicity and the risk of reversion to virulence (4).

To address this issue, researchers developed safer vaccine strains, such as nOPV2, which prevent the virus from regaining neurovirulence through a single mutation by stabilizing specific regions of the viral genome (e.g., domain V of the 5′ UTR) (45–47). Additionally, the cre element was repositioned to the 5′ UTR, and amino acid substitution mutations (D53N and K38R) were introduced in the RNA polymerase to reduce viral adaptability and recombination frequency, thereby further enhancing vaccine safety (47). Building on the success of nOPV2, researchers have also developed new vaccine candidate strains targeting *PV1* and *PV3* (nOPV1 and nOPV3). These candidate strains retain the antigenicity and immunogenicity of the Sabin strain while enhancing the stability of attenuated characteristics and reducing the likelihood of reversion to virulence. These vaccine strains exhibit good replication capacity in Vero cells and demonstrate higher attenuated virulence than traditional Sabin strains in transgenic mouse models (48). In the context of infectious bronchitis virus (IBV) in chickens, a similar attenuation process is employed. For instance, adaptive mutations that arise during the production of live attenuated vaccines against IBV often decrease virulence. A study identified a mutation at the 3′ end of the S gene in an IBV strain that was serially passaged in chicken embryos, resulting in a 9-aa truncation of the cytoplasmic tail (CT) of the S protein. This mutation significantly reduced the pathogenicity of the virus, underscoring the importance of such adaptive mutations in the attenuation process (49).

The cessation of serotype 3 OPV is a critical part of the polio endgame strategy. GPEI plans to conduct this in phases, with the cessation of serotype 2 OPV already completed. The cessation of OPV3 is expected to reduce cases of VAPP and minimize the risk of creating VDPVs, which pose a significant threat to eradication efforts (50). Thus, the attenuation process in OPV production is not only crucial for ensuring vaccine safety but also plays a pivotal role in global disease eradication strategies.

### Regulatory and safety considerations

#### WHO prequalification requirements for vaccine approval

The WHO prequalification program sets strict requirements for vaccine approval. For a vaccine to be prequalified, it must demonstrate pharmaceutical quality, safety, and efficacy. Pharmaceutical quality includes aspects such as the manufacturing process, stability, and purity of the vaccine. Safety is evaluated through extensive clinical trials, including the assessment of adverse events. Efficacy is determined by demonstrating the ability of the vaccine to induce a protective immune response.

For example, in the case of the trivalent influenza vaccine manufactured by Instituto Butantan, the WHO prequalification required the submission of a pharmacovigilance plan, including an active surveillance evaluation, and proof of a functional pharmacovigilance system at the institute (51).

#### Biosafety measures to prevent accidental release during production

Biosafety measures are of utmost importance during poliovirus vaccine production to prevent the accidental release of the virus. Facilities handling poliovirus must adhere to strict containment guidelines. These include the use of appropriate biosafety cabinets, proper waste management, and staff training on biosafety procedures.

For example, in the United States, the establishment of a Poliovirus Containment Program has been crucial. The program conducts site visits to verify the implementation of preliminary containment measures, such as primary containment, decontamination, hand hygiene, security, emergency response, training, and immunization practices at facilities retaining poliovirus (52).

#### Global supply chain dynamics and equitable distribution

The global supply chain for poliovirus vaccines faces several challenges in ensuring equitable distribution. The demand for vaccines, especially in resource-limited countries, often exceeds the supply. The production capacity of vaccine manufacturers is a limiting factor, and there can be delays in production and delivery. Additionally, the cost of vaccines, especially IPV, can be a barrier to access in some countries.

To address these issues, initiatives such as Gavi, the Vaccine Alliance, play a crucial role. Gavi aims to increase access to vaccines in developing countries by providing financial support and negotiating better prices with vaccine manufacturers (Table 1). However, challenges such as donor fatigue and the need for sustainable financing still need to be overcome to ensure a stable and equitable global supply of poliovirus vaccines (53).

**Table 1:** Major global manufacturers of polio vaccines.

Manufacturer	Product type	Location
Sanofi Pasteur	OPV, IPV	France
GlaxoSmithKline Biologicals	OPV, IPV	Belgium
Serum Institute of India	OPV, IPV	India
Bharat Biotech	OPV, IPV	India
Bilthoven Bio	OPV, IPV	Netherlands
Bio-Manguinhos/Fiocruz	OPV, IPV	Brazil
Beijing Institute of Biological Products Co., Ltd. (BIBP)	OPV, IPV	China
Hualan Biological Boviotech Co., Ltd.	OPV, IPV	China
Chengdu Institute of Biological Products Co., Ltd.	OPV, IPV	China

## Global deployment strategies and challenges

### Vaccination campaigns

#### Routine immunization vs. supplementary immunization activities (SIAs)

Routine immunization is the foundation of polio prevention, providing continuous protection to children. For example, only IPV has been used, with a routine immunization schedule of four doses administered at 2 months, 4 months, 6–18 months, and 4–6 years of age in the U. S. Most other countries in the Americas used OPV and introduced at least one dose of IPV. European countries widely used IPV as part of their routine immunization programs, usually in multiple doses. Some countries also combined OPV for specific populations or in specific situations. The routine immunization program in China includes multiple doses of bOPV and at least one dose of IPV. In India and some African countries, OPV is used in routine immunization programs, and at least one dose of IPV is introduced. However, routine immunization coverage is suboptimal in some areas, leaving populations vulnerable to poliovirus infection.

SIAs are conducted periodically to reach children who may have missed routine immunization doses, which boosts population immunity and keeps poliomyelitis rates low (54). The campaign is mainly implemented in some African countries, such as Egypt, Ethiopia, Nigeria, Congo, etc. (55). For example, India conducted large-scale polio SIAs, immunizing millions of children with OPV in 2009 (55). In Nigeria, the implementation of SIAs has significantly increased vaccination coverage and reduced the number of children missed through improved household-based microplanning (56). During the implementation of SIAs, significant improvements in vaccination coverage were achieved and the number of missed children was reduced through improvements in household-based micro-planning (57).

Additionally, countries in Eastern Europe and Central Asia have also conducted polio SIAs, such as Albania, Armenia, Azerbaijan, Belarus, Bosnia, Bulgaria, Croatia, Georgia, etc. (55). Middle Eastern countries such as Iran, Iraq, Lebanon, Libya, Syria, the United Arab Emirates, and Yemen have also conducted polio SIAs (55). Despite the challenges posed by the COVID-19 pandemic, SIAs were successfully implemented in Sabah, Malaysia, demonstrating their adaptability in crisis situations (58). Generally, SIAs serve as an effective strategy in increasing vaccine coverage and maintaining the global polio eradication goal. By continuously improving and adapting to the needs of different regions, SIAs have not only contributed to polio eradication but also provided valuable experience for the control of other vaccine-preventable diseases.

#### Cold chain logistics and oral vs. injectable vaccine delivery in low-resource settings

Cold chain logistics are crucial for maintaining the potency of poliovirus vaccines, especially in low-resource settings. Both oral and injectable vaccines require proper storage and transportation at specific temperatures. OPV is relatively more stable under certain conditions, but it still needs to be stored within a recommended temperature range.

In low-resource settings, challenges such as limited access to electricity for refrigeration and poor transportation infrastructure could affect the cold chain. Specifically, quality control of vaccine cold chain management is often neglected in resource-limited areas of Ethiopia. It was found that the overall magnitude of good knowledge and practice of vaccine cold chain management among health workers was 58% (95% CI: 52.2–64.3%) and 52.2% (95% CI: 46.3–58.4%), respectively (59), indicating the need for improvement. It was also found that health workers who had received pre-service training, had standard operating procedures/guidelines, and received supportive supervision performed better in terms of cold chain management knowledge (59). These factors significantly influenced the knowledge level and practical skills of health workers. Therefore, to improve the quality of vaccine cold chain management, it is imperative to strengthen the training and support of health workers in these resource-limited areas. Additionally, the delivery of injectable vaccines face challenges in terms of safety, as it requires trained personnel and proper disposal of needles and syringes.

### Eradication-endgame strategies

#### Synergistic use of IPV and OPV: transition from trivalent OPV (tOPV) to bivalent OPV (bOPV) and IPV

Trivalent oral poliovirus vaccine (tOPV) is composed of live attenuated poliovirus strains-Sabin 1, 2, and 3. The Sabin strains are genetically unstable and, in rare cases, can mutate and revert to neurovirulent strains, thereby causing VAPP in recipients or contacts of recipients. VAPP occurs when the attenuated poliovirus in OPV mutates into a neurovirulent variant during replication in the intestine, enters the central nervous system, and causes clinical symptoms indistinguishable from those caused by WPV paralysis. The Sabin type 2 component in tOPV is the primary cause of VAPP. It was reported that since the eradication of WPV2 in 1999, all poliomyelitis cases associated with PV2 have resulted from the continued use of tOPV. Sabin type 2 is responsible for approximately 40% of VAPP cases (60). Before switching to bivalent OPV (bOPV), tOPV was linked to up to 38% of all VAPP cases, or about 200 cases annually (61). According to a report, the risk of VAPP globally is approximately 4.7 cases per million births (range: 2.4–9.7), with an estimated annual global burden of 498 cases (range: 255–1,018). If only countries currently using OPV are considered, the VAPP risk is about 3.8 cases per million births (range: 2.9–4.7), with a burden of 399 cases annually (range: 306–490) (62). The epidemiological trends of VAPP vary by country income level. In low-income countries, most cases occur in individuals who have received more than three doses of OPV (63%), while in middle- to high-income countries, the majority of cases occur in individuals receiving their first OPV dose or in unvaccinated contacts (81%) (62). It was shown that in developing countries, tOPV is associated with rare cases of VAPP, occurring at a rate of approximately 2–4 cases per million births in a birth cohort (63). From 1980 to 2014, the U. S. reported 154 VAPP cases, all caused by the Sabin poliovirus strains in OPV vaccines (64).

The risk of VAPP is higher after the first dose of OPV, in communities with low vaccine coverage rates, and among immunocompromised individuals. The VDPVs cause paralysis with clinical signs and severity indistinguishable from those caused by WPV. On March 24, 2016, the WHO announced the implementation of the tOPV-to-bOPV transition process. Countries were required to gradually phase out tOPV and introduce bOPV by April 17, 2016, while also introducing IPV into their national immunization programs (61). The tOPV-to-bOPV transition was a critical step toward global polio eradication. It reduced the risk of VAPP and VDPV cases caused by the Sabin type 2 strain, bringing the world closer to achieving the goal of polio eradication. From April 17 to May 1, 2016, countries around the world successfully removed the Sabin 2 strain from OPV and introduced bOPV. The tOPV-to-bOPV transition was completed. Additionally, Multiple governments adjusted its immunization strategy to include one dose of IPV administered at 2 months of age, followed by two doses of bOPV at 3 and 4 months of age around 2017, such as China (2019), the U. S. (2017), the U. K. (2018), Australia (2018), and New Zealand (2017). After the switch to bOPV, the Sabin type 2 strain was removed from OPV, thereby eliminating the risk of VAPP caused by the Sabin type 2 strain. The number of VAPP cases caused by the Sabin type 2 strain began to decline significantly.

Specifically, six outbreaks of VDPVs occurred in four countries in 2016: Pakistan, Syria, Nigeria, and the Democratic Republic of Congo (65). In 2017 and 2019, 96 and 350 cases were reported, respectively (66). According to the WHO, a total of 528 cases of VDPVs were reported worldwide in 2023. Nigeria reported the highest number of cVDPV2 cases in 2024, accounting for 36%. A total of 288 cases of VDPVs have been reported worldwide in 2024. As of July 2025, 303 cases of VDPVs have been detected worldwide. No new cases of VAPP caused by the Sabin type 2 strain have been reported globally. Overall, the number of VDPVs cases worldwide has decreased compared to 2023, but the situation remains serious and requires continued strengthening of surveillance and immunization efforts.

The synergistic use of IPV and OPV is crucial during this transition phase. IPV, which contains inactivated virus, is administered to provide systemic immunity without the risk of VAPP. It is particularly effective in inducing a humoral immune response, which is essential for individual protection against poliovirus. On the other hand, bOPV, which contains live attenuated viruses, is used to maintain mucosal immunity in the gut, which is essential for interrupting virus transmission in the community. The combination of these vaccines aims to optimize both individual and herd immunity, thereby reducing the risk of poliovirus outbreaks (67). This approach aligns with WHO recommendations and is considered a reasonable option during the transition from OPV to IPV-only immunization schedules. The sequential use of IPV followed by bOPV may enhance the immunogenicity against poliovirus serotypes, providing a more robust defense against potential outbreaks (4, 67). Sequential IPV-OPV immunization schedules can induce a stronger immunogenic response compared to IPV alone. Research in India indicated that sequential administration of IPV and bOPV significantly reduced the excretion of poliovirus, indicating that IPV significantly enhance intestinal mucosal immunity (68). A randomized controlled trial in Chile demonstrated that IPV alone or in combination with bOPV sequential vaccination is feasible and effective in Chilean infants, with comparable immunogenicity and safety profiles, providing robust protection against poliomyelitis (69).

In conclusion, the transition from tOPV to bOPV and IPV is a key step in the global effort to eradicate polio. By leveraging the strengths of both IPV and OPV, health authorities can ensure a safer and more effective immunization strategy that minimizes the risks associated with live attenuated vaccines while maintaining prominent levels of community immunity. This strategic shift is essential for achieving and sustaining polio-free status in countries worldwide.

#### Containment of poliovirus stocks post-eradication (GAPIII guidelines)

After the eradication of wild polioviruses, the containment of poliovirus stocks is crucial to prevent reintroduction. The World Health Organization’s Global Action Plan III (GAPIII) provides guidelines for the containment of polioviruses in essential laboratory and vaccine production facilities. The GAPIII outlines critical steps for essential laboratory and vaccine production facilities that intend to retain materials confirmed to contain or potentially containing type-specific wild poliovirus, vaccine-derived poliovirus, or oral poliovirus vaccine/Sabin viruses. National authorities are tasked with certifying that these facilities meet the containment requirements described in GAPIII, which is beneficial for preventing the reintroduction of poliovirus into a polio-free world (70).

As of August 2019, 26 countries have nominated 74 poliovirus-essential facilities (PEFs) to retain PV2 materials. These facilities are required to meet stringent containment criteria, including bio-risk management requirements. National authorities for containment (NACs) have been established in many countries to oversee the certification process. Failure to contain poliovirus stocks properly could lead to the reemergence of the disease, as seen in some historical incidents of facility-associated release of polioviruses (71).

### Case studies

#### Success stories: India’s polio-free certification

India’s polio-free certification in 2014 marked a monumental milestone in public health, not only for the country but also for the global fight against poliomyelitis. This achievement was the result of decades of concerted efforts, strategic planning, and relentless execution of vaccination campaigns. The journey from being a polio-endemic country to achieving polio-free status involved overcoming numerous challenges, including high population density, diverse geographical terrains, and socio-economic barriers.

One of the critical strategies that contributed to India’s success was the implementation of extensive pulse polio vaccination campaigns. These campaigns were conducted multiple times a year, ensuring that every child under the age of five received the OPV. The introduction of monovalent and bivalent OPV, which were more effective against specific strains of the poliovirus, played a crucial role in interrupting the transmission of the virus in high-risk areas like Uttar Pradesh and Bihar (72).

The involvement of civil society and community mobilization was another pivotal factor. Initiatives like the CORE Group Polio Project (CGPP) helped bridge the gap between communities and government programs by addressing the concerns of families who were initially resistant to vaccination. This grassroots approach ensured higher vaccination coverage and built trust within communities (73).

The economic and health benefits of polio elimination in India have been substantial. It is estimated that the national polio program averted millions of cases of paralytic polio and saved hundreds of thousands of lives. The economic productivity gains from these efforts have been valued at trillions of dollars, highlighting the significant return on investment in public health initiatives (74).

The role of social mobilization networks, such as the Social Mobilization Network (SMNet), cannot be overstated. These networks were instrumental in reaching hard-to-reach populations and converting vaccine-resistant families into advocates for polio immunization. The success of these networks demonstrates the power of community engagement in public health campaigns (75).

Finally, the introduction of the IPV into India’s national immunization schedule was a strategic move to sustain polio-free status. IPV provides complete individual protection and is essential in the post-eradication era to prevent the re-emergence of the virus (76).

India’s journey to becoming polio-free serves as an inspiring example for other countries battling similar public health challenges. The lessons learned from India’s experience can inform global strategies for eradicating other vaccine-preventable diseases and achieving broader public health goals.

#### Challenges in conflict zones and areas with vaccine hesitancy

In conflict zones, such as parts of Afghanistan and Pakistan, polio vaccination faces numerous challenges. Insecurity makes it difficult to reach children with vaccines, and there may be active opposition to vaccination efforts. In areas with vaccine hesitancy, misinformation and cultural beliefs lead to low vaccination coverage. Rumors about the safety and efficacy of the polio vaccine have spread, resulting in parents refusing to vaccinate their children (77). The GPEI has made substantial progress in vaccinating millions of children worldwide, including those living in communities affected by conflicts and other humanitarian emergencies. However, the persistence of indigenous WPV transmission in these areas continues to threaten the success of global polio eradication efforts (78).

One of the primary obstacles to polio vaccination in conflict zones is the disruption of health care and immunization services. The ongoing violence and insecurity make it difficult for health workers to reach vulnerable populations, leaving many children without access to lifesaving vaccines. This situation is exacerbated by the displacement of populations, which further complicates the delivery of immunization services. In the Middle East, for instance, a polio outbreak occurred in Syria in 2013, two years after the onset of the Syrian civil war, highlighting the vulnerability of conflict-affected regions to polio outbreaks due to disrupted health services (79).

Efforts to address these challenges have included the implementation of innovative strategies to increase vaccine coverage. In Pakistan, for example, community engagement and integrated health and polio immunization campaigns have been employed to strengthen community buy-in and enhance immunity through the introduction of IPV in combination with OPV. These strategies have shown success in increasing vaccine coverage, even in high-risk, conflict-affected areas, demonstrating the importance of community mobilization and targeted health interventions in overcoming the barriers to polio vaccination in conflict zones (80).

## Emerging challenges

### Vaccine-derived polioviruses (VDPVs)

#### Strategies for outbreak response

Rapid response to VDPVs outbreaks is crucial to prevent the spread of the virus. The deployment of nOPV2 has been an important strategy. The World Health Organization’s Prequalification program issued an Emergency Use Listing (EUL) recommendation for nOPV2 in 2020, enabling its use in outbreak response. In Nigeria, the use of nOPV2 in response to VDPVs outbreaks has been evaluated. Modeling studies suggest that using nOPV2 intensively, along with other measures such as early detection and high-coverage vaccination campaigns, can substantially increase the probability of ending VDPVs transmission globally. However, challenges such as limited vaccine supply and the need for rapid deployment in affected areas still need to be addressed (81).

### Funding and political barriers

#### Donor fatigue and sustaining financial commitments post-COVID-19

The COVID-19 pandemic had an impact on donor fatigue and the sustainability of financial commitments for polio eradication. With the diversion of resources to combat the COVID-19 pandemic, there is a risk of reduced funding for polio eradication efforts. Donor fatigue may also occur due to the long-term nature of the polio eradication program and the perceived slow progress in some areas.

For example, in some countries, the suspension of polio supplementary immunization activities during the COVID-19 pandemic led to a decline in vaccination coverage, increasing the risk of polio outbreaks. Sustaining financial commitments from donors is crucial to continuing the efforts to interrupt wild poliovirus transmission and address the challenges posed by VDPVs (82).

Moreover, the pandemic-induced disruptions in health services, including immunization campaigns, have further complicated the financial landscape for polio eradication. The GPEI faced substantial challenges in maintaining its momentum due to the intentional and unintentional reductions in health services, which included immunization efforts against WPV and VDPVs. These disruptions necessitated a strategic response to mitigate the impact of COVID-19 on polio eradication efforts. As the GPEI resumed its activities, it had to navigate the dual challenges of addressing the backlog of immunizations and securing the necessary financial resources to sustain its operations in a post-pandemic world (83).

The pandemic has underscored the importance of resilient funding mechanisms that withstand global health crises. As the world continues to grapple with the aftermath of COVID-19, it is necessary for stakeholders involved in polio eradication to reassess and reinforce their financial strategies. This includes engaging with donors to renew their commitments and exploring innovative funding models that can ensure the continuity of eradication efforts. By addressing donor fatigue and securing sustainable financial commitments, the global community can continue to make strides toward the ultimate goal of eradicating polio (64).

#### Geopolitical obstacles in endemic regions

Geopolitical obstacles in endemic regions, such as Afghanistan and Pakistan, pose significant challenges to polio eradication. Geopolitical tensions lead to a lack of coordination between neighboring countries, which is essential for effective cross-border vaccination campaigns. These obstacles need to be addressed through diplomatic efforts and international cooperation to ensure the successful eradication of polio in these regions (84).

The cross-border transmission of WPV between Afghanistan and Pakistan remains a significant concern. Both countries are considered a single epidemiologic block, and the movement of populations across their shared border complicates efforts to interrupt virus transmission. Despite efforts to improve cross-border coordination and surveillance, the continuous movement of people poses a risk of reintroducing the virus into areas where it has been previously controlled (85).

Moreover, the distrust of vaccination programs, fueled by misinformation and historical events, has led to vaccine hesitancy and refusals in these regions. For instance, the use of a fake vaccination campaign by the CIA in Pakistan to gather intelligence has had long-lasting effects on public perception, leading to increased suspicion and resistance to polio vaccination efforts (86). This distrust is compounded by cultural and religious beliefs, which sometimes conflict with public health initiatives, making community engagement and education critical components of the eradication strategy (87).

Efforts to overcome these challenges have included the implementation of innovative strategies such as engaging local leaders and community members to build trust and improve vaccination coverage. Additionally, the recruitment of female health workers has been crucial in reaching women and children in conservative communities where male health workers may not be allowed (88). Despite these efforts, achieving high vaccination coverage in all areas remains a formidable task.

To address these geopolitical challenges, sustained political commitment and increased accountability at all levels are essential. This includes enhancing the quality of SIAs and routine immunization services, as well as improving the oversight and management of polio eradication programs. By addressing the underlying reasons for community resistance and operational issues, it is possible to enhance the effectiveness of vaccination campaigns and move closer to the goal of global polio eradication (89, 90).

### Immunization gaps

#### Impact of the COVID-19 pandemic on polio vaccination coverage

The COVID-19 pandemic has had a significant impact on polio vaccination coverage. The suspension of routine immunization services and SIAs during the pandemic led to a decline in vaccination coverage in many countries. A global assessment by the GPEI found that the number of acute flaccid paralysis (AFP) cases reported declined 33% between January–September 2020 compared to the same period in 2019, indicating potential gaps in vaccination coverage (91).

#### Addressing inequities in access to IPV

Inequities in access to IPV also exist. High-income countries may have better access to IPV due to their financial resources and infrastructure, while low-and middle-income countries may face challenges in obtaining sufficient supplies. Addressing these inequities requires international cooperation, such as through initiatives like Gavi, to ensure that all countries have access to the necessary vaccines for polio prevention.

## Future directions

### Innovations in vaccine technology

#### Thermostable formulations, microneedle patches, and nanoparticle-based delivery

Thermostable formulations of poliovirus vaccines are being developed to reduce the reliance on the cold chain. For example, dissolving microneedle (MN) patches for IPV have been developed. These patches, formulated with maltodextrin and D-sorbitol in histidine buffer, can maintain > 70% activity after 2 months and > 50% activity after 1-year storage at 5°C or 25°C with desiccant (92).

Microneedle patches offer several advantages, including pain-free administration, self-administration potential, and reduced risk of needle-stick injuries. Nanoparticle - based delivery systems can enhance the immunogenicity of vaccines by improving antigen delivery to immune cells. For example, up conversion nanoparticle-powered microneedle patches have been developed for transdermal delivery of siRNA, showing potential for improving vaccine delivery (93).

#### Universal poliovirus vaccines to address all serotypes

IPV is made from inactivated viruses and contains viral components from all three serotypes. It stimulates the body to produce humoral immunity (antibodies) against all three serotypes, thereby providing protection. OPV is made from attenuated live viruses and can be classified as tOPV or bOPV (targeting specific serotypes) depending on the serotypes it contains. Theoretically, tOPV covers all three serotypes. However, they still have limitations, prompting scientists to explore more ideal “universal” vaccines.

As mentioned above, OPV has the potential to restore pathogenicity in extremely rare cases. Developing a “universal” vaccine with no such risk (such as a new mRNA vaccine or subunit vaccine) should be a future breakthrough. Due to differences in antigenicity and infectivity among the three serotypes of the virus, the immune response elicited by IPV and OPV may vary between serotypes. For example, OPV typically provides better immunity against *PV1* and *PV3*, but may be relatively less effective against *PV2* (which is why, even after *PV2* was declared eradicated in 2015, many countries still need to retain the *PV2* component in their vaccines to prevent recurrence). Polio viruses could mutate, causing the antigenicity of existing vaccines to mismatch with the antigenicity of the virus that’s actually spreading (aka “antigenic drift”). One key way to deal with this risk is to develop a “universal” vaccine with broader coverage against virus variants. Interestingly, the current IPV and OPV require multiple doses and adjustments to the combination (such as trivalent or bivalent) based on regional prevalence. Developing a vaccine covering all serotypes in a single dose without the requirement for combination adjustments would simplify immunization programs greatly.

Most excitingly, new technologies are rapidly developing. “Universal” vaccines developed using these technologies may play a key role in future eradication programs. A study reported a novel low-cost, cold chain-free booster vaccine using poliovirus capsid protein (VP1) fused with cholera non-toxic B subunit (CTB) expressed in lettuce chloroplasts. Mice primed with IPV and boosted with this plant - based vaccine showed enhanced VP1-specific IgG1, VP1-IgA titer, and neutralization against all three Sabin serotypes (94). Another approach is the use of human monoclonal antibodies that can neutralize all three serotypes. A study identified a human monoclonal antibody 9H2 that binds within the poliovirus receptor-binding site to neutralize all three serotypes, providing a potential basis for the development of antiviral (95).

It has been reported that tobacco plants were able to express and assemble particle structures similar to norovirus. Animal experiments have shown that plant-derived norovirus-like particle vaccines induced strong immune responses in the body, including the production of high levels of specific antibodies and the activation of cellular immune responses, indicating good immunogenicity of the vaccine (96). Similar investigations have also been conducted in the field of polio vaccines. Researchers produced virus-like particles (VLPs) targeting *PV3* from plants. The vaccine induced an immune response similar to natural poliovirus in animal experiments, indicating that they have strong immunogenicity and effectively activate the body’s humoral immune response (97). Most recently, the carrot (*Daucus carota*) cell suspension system SMC-1 has been reported for use in the production of VLPs. Successful expression of virus-like particles was achieved by introducing genes encoding poliovirus capsid proteins into carrot cells. These VLPs were structurally similar to real poliovirus and elicited an immune response (98). The researchers constructed a carrot cell line (SMC-1) to express the VP2 antigen of poliovirus at both callus and cell suspension levels. The effects of different culture media (MS and B5), urea concentration, plant growth regulators (2,4-D and KIN), and light conditions (continuous light, photoperiod, and complete darkness) on cell growth and antigen expression were investigated. It was shown that SMC-1 suspension cell line based on a bioreactor had a higher production rate for VP2 protein compared with flask culture, providing a key perspective to produce low-cost polio vaccines. Consequently, vaccine antigens generated by plant expression systems exhibit excellent characteristics in terms of stability and immunogenicity and offer a promising and effective vaccine option for the prevention and control of poliomyelitis, thereby further advancing efforts to eradicate the disease.

### Strengthening global collaboration

#### Role of public-private partnerships

Public-private partnerships play a key role in polio eradication. Gavi, the Vaccine Alliance, and the Bill & Melinda Gates Foundation have been actively involved in supporting vaccination programs in developing countries. Gavi provides financial support, negotiates with vaccine manufacturers for better prices, and helps in the distribution of vaccines.

The Gates Foundation has funded research and development efforts, as well as capacity-building projects. For example, in Nigeria, the Gates Foundation - funded Partnership for Advocacy in Child and Family Health Project (PACFaH) has helped in building the capacity of indigenous NGOs for social accountability in child and family health, including polio vaccination (99).

#### Integration of polio eradication with broader health systems

Integrating polio eradication efforts with broader health systems is essential for long-term sustainability. The Global Polio Eradication Initiative has built significant infrastructure and capacity in many countries, including surveillance systems, trained personnel, and cold chain facilities. These assets can be used to strengthen routine immunization and other health programs.

A study in the Eastern Mediterranean region found that a project aimed at strengthening public health capacity for polio eradication and routine immunization activities helped in building effective surveillance and immunization systems, as well as improving the capacity to control other vaccine - preventable diseases (100).

### Post-eradication preparedness

#### Surveillance for poliovirus resurgence

Surveillance for poliovirus resurgence is crucial to detect any potential re - introduction of the virus. Environmental surveillance, in addition to acute flaccid paralysis surveillance, can help in early detection. For example, in Japan, environmental surveillance during the transition from OPV to IPV detected the excretion of polioviruses from the immunized population (101).

#### Ethical considerations for phasing out OPV and maintaining IPV stockpiles

Ethical considerations for phasing out OPV and maintaining IPV stockpiles are complex. Phasing OPV is necessary to prevent the emergence of VDPVs, but it requires careful planning to ensure that the population remains protected. Maintaining IPV stockpiles is important for outbreak response, but decisions need to be made regarding the appropriate quantity and distribution of the stockpiles, considering factors such as cost, storage, and access.

## Conclusion

Since the launch of the Global Polio Eradication Initiative in 1988, significant milestones have been achieved. The number of wild poliovirus cases has decreased by over 99%, with wild poliovirus type 2 eradicated in 1999 and wild poliovirus type 3 not detected since 2012 (102). Many countries, including India, have been certified polio-free. However, several unresolved issues remain. Endemic transmission in Afghanistan and Pakistan persists, and the emergence of VDPVs continues to pose a threat. Challenges in vaccine production, such as ensuring consistent quality and scaling up, and issues in vaccine distribution, including equitable access and maintaining the cold chain, also need to be addressed.

Sustained political will is essential for the successful eradication of polio. Political leaders need to prioritize polio eradication, allocate sufficient resources, and ensure the cooperation of all stakeholders. Scientific innovation is also crucial, such as the development of new vaccines, improved production methods, and better delivery systems. Equitable resource allocation is necessary to ensure that all countries, especially those in resource-limited settings, have access to vaccines and related services. This requires international cooperation, with high-income countries supporting low- and middle-income countries in their polio eradication efforts.

The vision of a polio-free world is within reach, but it requires continued efforts. The lessons learned from the polio eradication initiative can be applied to future disease eradication efforts. These include the importance of strong global partnerships, the need for a comprehensive approach that combines vaccination, surveillance, and community engagement, and the significance of addressing social and political barriers. For example, the experience of building partnerships and coordination mechanisms in India during polio eradication could be replicated in other disease control programs. Additionally, the challenges faced in polio eradication, such as dealing with vaccine hesitancy and ensuring equitable access, can inform the strategies for future disease eradication initiatives.
